# The Mitigatory Effect of Shen-Qi Compound on the Diabetic Thoracic Aortic Complications through Inhibiting the Inflammatory Microenvironment by miR-223-3p/RBP-J/IRF8 Axis

**DOI:** 10.1155/2022/6686931

**Published:** 2022-09-28

**Authors:** Ye Tian, Gang Xu, Hong Gao, Hong-Yan Xie, Yu-Lin Leng, Xiao-Xu Fu, Chun-Guang Xie

**Affiliations:** TCM Regulating Metabolic Diseases Key Laboratory of Sichuan Province, Hospital of Chengdu University of Traditional Chinese Medicine, Chengdu, China

## Abstract

**Background:**

Disruption of the vascular immunological inflammatory microenvironment is linked to metabolic memory impairment. Even though it has been proven that the Shen-Qi compound (SQC) can efficiently halt metabolic memory and preserve vascular endothelial cells, extensive studies need to be done to investigate if it can also change the vascular immune-inflammatory microenvironment by regulating the immune system. This will help figure out the role of stopping metabolic memory.

**Methods:**

After 4 weeks on a high-fat diet (HFD), GK rats were used to create a model for diabetic thoracic aortic problems. The effect and mechanisms of SQC on diabetic thoracic aortic complications were assessed by hematoxylin-eosin (H&E) staining, enzyme-linked immunosorbent assay (ELISA), biochemical analysis, terminal deoxynucleotidyl transferase deoxyuridine triphosphate (dUTP) nick end labeling (TUNEL), reverse transcription, real-time polymerase chain reaction (RT-qPCR), immunofluorescence (IF), western blot, and luciferase reporter assays.

**Results:**

SQC treatment ameliorates the HFD-induced pathological symptoms as well as the HFD-induced increased concentrations of fasting blood glucose (FBG), fasting insulin (FINS), total cholesterol (TC), triglycerides (TGs), and low-density lipoprotein cholesterol (LDL-C) and decreased concentrations of high-density lipoprotein cholesterol (HDL-C). Besides, SQC counteracted the HFD-induced average fluorescence intensity of vascular cell adhesion molecule-1 (VCAM-1) and intercellular adhesion molecule-1 (ICAM-1), as well as the concentrations of endothelin-1 (ET-1) and monocyte chemoattractant protein-1 (MCP-1), while rescuing the HFD-induced concentrations of nitric oxide (NO) and nitric oxide synthetase (NOS). Also, SQC decreases apoptosis and oxidative stress in rats with diabetic thoracic aortic complications. In addition, SQC facilitated the polarization of macrophages, stimulated the activation of dendritic cells, and regulated the inflammatory milieu in rats with diabetic thoracic aortic complications. Furthermore, SQC also modulated the miR-223-3p/RBP-J/IRF8 axis in the macrophages of rats with diabetic thoracic aortic complications.

**Conclusion:**

SQC ameliorated diabetic thoracic aortic complications through the regulation of apoptosis, oxidative stress, and inflammatory microenvironment mediating by the miR-223-3p/RBP-J/IRF8 axis.

## 1. Introduction

Diabetes is one of the most common and grievous metabolic diseases and has been estimated at approximately 463 million adult patients worldwide based on the International Diabetes Federation. Moreover, the incidence of diabetes has sharply increased, with an estimation of 700 million diabetic patients by 2045 [1]. Moreover, diabetes is closely associated with multiple morbidity- and mortality-causing consequences [2]. Diabetic macrovascular disease, also known as diabetic thoracic aortic disease, is one of the most prevalent complications of diabetes, accounting for about 60–70% of the causes of diabetes-related deaths [3]. According to the Guidelines for the Prevention and Treatment of Diabetes in China, the incidence of diabetic thoracic aortic complications has increased significantly. In China, it has become a chronic complication with the highest mortality and morbidity rates, causing the most damage. Based on multiple prospective clinical trials, the metabolic memory phenomenon has been identified as one of the causes of diabetic thoracic aortic complications. This is the idea that metabolic disturbances caused by hyperglycemia to target organs in the early stages can cause permanent damage and make complications worse [4, 5]. Although oxidative stress is a key factor in triggering and maintaining metabolic memory [6], the clinical application of antioxidants to intervene in metabolic memory and treat diabetic thoracic aortic complications is not satisfactory. In this view, finding effective measures to control and block metabolic memory that delay the occurrence and development of diabetic thoracic aortic complications is of great social and economic significance.

Furthermore, multiple studies have demonstrated that inflammation is involved in diabetes and associated complications. Diabetes and its complications are inflammations mediated by nonspecific immune mediators such as inflammatory cells and their secreted inflammatory factors and some acute response substances that belong to the innate immune response [7]. One important cause of diabetic thoracic aortic complications is that high blood sugar constantly causes the vascular endothelium to make glucotoxic products, change or disrupt cell signaling pathways, and cause vascular immune cells to gather and start an inflammatory response. Thus, diabetic thoracic aortic complications are a low-grade inflammatory state that is also a chronic immune-inflammatory response. Cell groups mainly composed of monocytes, macrophages, and dendritic cells scatter under the normal arterial intima, which is responsible for monitoring the microenvironment in which they are located to find potentially harmful antigens and then to maintain a balance. When inflammatory factors and other harmful antigens invade, it will break the balance, activate lymphocytes, cause a series of specific immune responses, trigger persistent inflammatory immune responses, and lead to immune-inflammatory microenvironment disorders [8]. Moreover, it has been shown that metabolic memory impairment is closely linked to the disruption of the vascular immune-inflammatory microenvironment. Hence, clearing harmful antigens such as inflammatory factors and balancing immune cells can regulate immune-inflammatory microenvironment disorders and reduce vascular damage, which contributes to the prevention and treatment of diabetic thoracic aortic complications.

Previous studies have shown that qi and yin deficiency accompanied by blood stasis are the main syndromes of diabetic thoracic aortic complications [9]. Thus, the nourishing yin, benefiting qi, and transforming stasis method has been demonstrated to be effective in the treatment of the diabetic thoracic aortic complications [10–12]. Shen-Qi compound created according to the nourishing yin, benefiting qi, and transforming stasis method has positive effects on regulating blood lipids, blood sugar, insulin resistance, and blood flow and repairing vascular endothelial damage, which can effectively overcome the development and occurrence of diabetic thoracic aortic complications [13, 14]. The Shen-Qi compound has also been shown to effectively block metabolic memory and improve the complications of diabetes in the thoracic aorta [15]. However, whether it can affect the vascular immune-inflammatory microenvironment by regulating the immune mechanism to clarify the role of suppressing metabolic memory still needs to be further explored.

Herein, the effect and mechanism of the Shen-Qi chemical on rats with diabetic thoracic aortic complications were examined. The goal of the underlined study is to provide an academic foundation for the development of a candidate therapy against thoracic aorta issues in diabetics.

## 2. Materials and Methods

### 2.1. Animals

Adult male GK rats (220–240 g) and adult male Wistar rats (200–210 g) were provided by SLRC Laboratory Animal Co. Ltd., Shanghai, China. Both rats were fed with water ad libitum with 55%–61% relative humidity and a 12-h/12-h light-dark cycle at 22 ± 1°C in an SPF room. All the ethical standards were approved by the ethical committee of the Chengdu University of Traditional Chinese Medicine (ID: 2021DL-011).

### 2.2. Groups and Treatments

After being adapted to the SPF laboratory conditions for a week before experiments, the FBG in all rats that were fasted for eight hours was detected by a blood glucose meter (Johnson & Johnson Medical Devices Companies, Shanghai, China). GK rats with an FBG <11.1 mmol/L and Wistar rats with FBG >5.6 mmol/L were excluded. Then, the GK rats were randomly divided into 5 groups according to the blood sugar level (*n* = 6), including Model, Model + MetHCl, Model + SQC-low, Model + SQC-middle, and Model + SQC-high, while the same numbers of Wistar rats were used as control rats. A diabetic thoracic aortic disease was developed using GK rats that were continuously fed a high-fat diet consisting of 88.2% general animal feed combined with 10% refined lard, 1.5% cholesterol, and 0.3% pig bile salts (HFK Bioscience Co., LTD, Beijing, China). The animals were housed in a vacuum-packed condition against light and humidity for 4 weeks. The underlined model can accurately simulate the early process of metabolic memory, which was confirmed by our lab. Wistar rats were fed with a normal chow diet, which consisted of 71% carbohydrates mixed with 4.5% fat and 20% protein. After 4 weeks of modeling, the GK rats in Model + MetHCl, Model + SQC-low, Model + SQC-middle, and Model + SQC-high were received by gavage of 0.1 g/kg/d metformin (Bristol Myers Squibb, Shanghai, China), 7.2 g/kg/d Shen-Qi Compound (SQC), 14.4 g/kg/d SQC, and 28.8 g/kg/d SQC, respectively; while the rats in Model and Control groups were fed by gavage of 5.0 mL/kg/d sterile pure water for 12 weeks at 8 : 00–10 : 00 am daily. SQC that functions as nourishing yin, nourishing qi, and promoting blood circulation was composed of 15 g ginseng (Lot: 1801074), 15 g *Astragalus* (Lot: 1805070), 10 g of *Rehmannia glutinosa* (Lot: 1806029), 10 g *Cornus* (Lot: 1806028), 10 g Chinese yam (Lot: 1804032), 10 g *Salvia* (Lot: 1805047), 10 g *Trichosanthes* (Lot: 1710067), and 6 g *rhubarb* (Lot: D1803026) (all from Chengdu New Lotus Chinese Medicine Co., Ltd., Sichuan, China), which was prepared by the Pharmacy Department of the Sichuan Provincial Hospital of Traditional Chinese Medicine. The SQC used in our study was an extractum, which was made as follows: ginseng was ground into a coarse powder and then soaked in 30% ethanol for 24 h. Next, the ethanol immersion solution was added at a rate of 3 ml/min until the liquid became colorless. Then, the liquid was collected and allowed to rest for 24 h. The supernatant was filtered and concentrated to a thick paste. The ginseng filter residue and the other seven flavors were boiled twice in water and then filtrated to collect the filtrate. The collected filtrate was mixed with the thick paste of ginseng to concentrate it into a thick paste. After an intervence for 12 weeks, the rats were intraperitoneally injected with 20% urethane according to the standard of 1 g/kg body weight to collect the blood and tissue samples.

### 2.3. Histological Analysis

Tissues from the thoracic aorta were separated, embedded in 4% formaldehyde, dehydrated, and cut into pieces. Hematoxylin and eosin (H&E) were then used to stain the tissue sections. The stained sections were photographed using an Olympus light microscope (Tokyo, Japan), and the program Image-Pro Plus 6.0 (Media Cybernetics, USA) was used to analyze the recorded images.

### 2.4. Enzyme-Linked Immunosorbent Assay (ELISA)

The serum levels of fasting insulin (FINS), interleukin (IL)-6, interferon (IFN)-*γ*, tumor necrosis factor (TNF)-*α*, IL-4, transforming growth factor (TGF)-*β*1, IL-10, IL-17A, nitric oxide synthases (NOS), endothelin-1 (ET-l), monocyte chemoattractant protein-1 (MCP-1), and oxidized low-density lipoprotein (ox-LDL) were examined by mouse Rat FINS ELISA kit (ZC-54525), Rat IL-6 ELISA kit (ZC-36404), Rat IFN-*γ* ELISA kit (ZC- 36294), Rat TNF-*α* ELISA kit (ZC-37624), Rat IL-4 ELISA kit (ZC-36402), Rat TGF-*β*1 ELISA kit (ZC-37645), Rat IL-10 ELISA kit (ZC-36379), Rat IL-17A ELISA kit (ZC-36387), Rat NOS ELISA kit (ZC-37503), Rat ET-1 ELISA kit (ZC-37024), Rat MCP-1 ELISA kit (ZC-36497), and Rat ox-LDL ELISA kit (ZC-37496; all from ZCIBIO Technology Co., Ltd., Shanghai, China) based on the manufacturer's instructions. In addition, the levels of ox-LDL in the aorta were detected by the Rat ox-LDL ELISA kit (ZC-37496; ZCIBIO Technology Co., Ltd.). The absorbance at 450 nm of wells was measured using a microplate reader (SpectraMAX Plus384; Molecular Devices, Shanghai, China).

### 2.5. Biochemical Analysis

An automatic biochemical analyzer (model 7150; Hitachi, Tokyo, Japan) was used to measure the serum levels of TC, TG, LDL-C, and HDL-C following the manufacturer's instructions. Additionally, the NO detection kit (A012-1-2) and glucose detection kit (A145-1-1, both from Nanjing Jiancheng Bioengineering Institute, Nanjing, China) were used to measure the levels of NO and glucose following the instructions in the operating manual.

### 2.6. Terminal-DeoxynucleotidylTransferase-Mediated Nick End Labeling (TUNEL) Staining

Apoptosis in endothelial cells from rat thoracic aorta tissues was evaluated by TUNEL staining. Briefly, the paraffin sections (5 *μ*m) were subjected to an in situ cell death detection kit (49330900; Roche Applied Science, Basel, Switzerland) according to the manufacturer's instructions. Images were captured with confocal microscopy (LSM700; Zeiss, Oberkochen, Germany). The apoptotic index was exhibited as the ratio of the number of TUNEL-positive cells to the total number of cells.

### 2.7. Reverse Transcription-Quantitative Polymerase Chain Reaction (RT-qPCR) Analysis

Total RNA from spleen tissues was extracted using RNA TRIzol Reagent (TaKaRa Biotechnology Co., Ltd., Dalian, China), and RT was performed using the PrimeScript RT reagent Kit (TaKaRa) based on the manufacturer's instructions. RT-qPCR was conducted in a 20 *μ*l mixture, including 2 *μ*l of the cDNA templates, 10 *μ*l 2 × TB Green™ Premix Ex Taq™ II (Tli RNaseH Plus; TaKaRa), 0.8 *μ*l of the 10 *μ*M forward and reverse primers, and 6.4 *μ*l ddH_2_O, using PikoReal 96 (Thermo Fisher Scientific). The RT-qPCR conditions were as follows: 5 min at 95°C, followed by 45 cycles between 95°C for 5 s and 55°C for 30 s, and 72°C for 30 s. The relative mRNA expressions of CD86, CD206, ROR-*γ*t, Foxp3, T-bet, and GATA3 were analyzed using the 2^−ΔΔCT^ method and normalized to the housekeeping gene *β*-actin. For the quantification of miR-223-3p expression, the RT reaction was carried out by Bulge-Loop™ miRNA RT-qPCR Primer (RiboBio Co., Ltd., Guangzhou, China). The RT reaction was performed at 42°C for 60 min and 70°C for 10 min. Gene expression levels were analyzed at 95°C for 10 min, followed by 40 cycles at 95°C for 2 s, 60°C for 20 s, and 70°C for 10 s. U6 acted as the housekeeping gene. The primers used in the current study are listed in Table 1.

### 2.8. Immunofluorescence (IF) Assay

After dewaxing, paraffin sections were treated with citrate buffer (pH = 6) to retrieve antigens. Then, sections were rinsed with phosphate buffer saline (PBS; Beyotime, Shanghai, China) thrice and blocked with goat serum (SP9002; ZSGB-BIO, Beijing, China) for 20 min at room temperature. Next, the sections were incubated with the iNOS antibody (1 : 100, ab178945; Abcam, Cambridge, UK), CD206 antibody (1 : 100, 24595; Cell Signaling Technology, Inc., Danvers, MA, USA), HLA-DR antibody (1 : 100, MA5-32232; Thermo Fisher Scientific, Waltham, MA, USA), CD11c antibody (1 : 100, A1508; ABclonal, Wuhan, China), ICAM-1 antibody (1 : 100, 10020-1-AP; Protein Tech, Wuhan, China), and VCAM-1 antibody (1 : 100, ab134047; Abcam) overnight at 4°C. Subsequently, the sections were washed with PBS three times and incubated with FITC-conjugated Goat Anti-Rabbit IgG (H + L) (1 : 100, GB22303; Servicebio, Wuhan, China) or Cy3-conjugated Goat Anti-Rabbit IgG (H + L) (1 : 100, GB21303; Servicebio) at 37°C for 30 min. The nuclei were stained with DAPI (ZLI-9557; ZSGB-BIO) for 10 min at room temperature. Images were captured using a confocal microscope (LSM700; Zeiss, Oberkochen, Germany).

### 2.9. Collection of Peritoneal Macrophages

After two lavages, the peritoneal lavage fluid was collected and centrifuged by density centrifugation at 4°C and 2000*g* for 15 min, and the supernatant was discarded. If there were numerous red blood cells after centrifugation, two to three times the volume of red blood cell lysate (Beyotime) was added, mixed, and centrifuged again after resting for two minutes. After discarding the supernatant, DMEM complete culture medium (Beyotime) including 10% fetal bovine serum (Beyotime) and 1% penicillin-streptomycin (Beyotime) was added, resuspended, and inoculated into 12-well plates for culture for 4 h. The medium was changed after the macrophages adhered, and the macrophages were collected by trypsinization (Beyotime) after culturing for 24 h.

### 2.10. Western Blot Analysis

Total proteins were obtained from macrophages and thoracic aortas via RIPA lysis buffer (Boster, Wuhan, China) and quantified using the BCA kit (P0009; Beyotime). Then, protein samples were separated by 10% SDS-PAGE and electrically transferred onto PVDF membranes (EMD Millipore, Billerica, MA, USA). The membranes were blocked in 5% skim milk (Anchor, Switzerland) for 2 h at room temperature and subsequently incubated with primary antibodies (ROR-*γ*t (1 : 2000, bs-23110R; Bioss), Foxp3 (1 : 2000, A12051; ABclonal), T-bet (1 : 1000, A4682; ABclonal), GATA3 (1 : 2000, A1638; ABclonal), RBP-J (1 : 2000, A5675; ABclonal), IRF8 (1 : 2000, DF13627; Affinity), (IFN-*γ*,1:12000,15365-1-AP;proteintech),(IL-4,1:12000,A14660;abclonal), (IL-6,1:1000,BS-4539R;bioss),(IL-10,1:2000,A2171;abclonal),(IL-17A,1:2000,A12454;abclonal), (TGF-*β*1,1:1000,BS-20411R;bioss),(TNF-*α*,1:2000,A0277;abclonal) and *β*-actin (1 : 100000, AC026; ABclonal)) overnight at 4°C. After being washed with PBS three times, the membranes were hatched with the corresponding secondary antibody for 3 h at room temperature. Protein expressions were analyzed relative to *β*-actin. Bands were visualized using an ECL chemiluminescence kit (EMD Millipore) according to the operating manual. The gray value was assessed by Image-Pro Plus software (Media Cybernetics, Inc., Rockville, MD, USA).

### 2.11. Dual-Luciferase Reporter Assay

The possible binding between miR-223-3p and RBP-J was predicted by the TargetScan website (https://www.targetscan.org/vert_71/). The WT and mutant RBP-J 3′UTRs were inserted into the pmiRGLO vector (Promega, Madison, WI, USA). The miR-223-3p-mimics or specified luciferase reporter vectors were transfected into macrophages. After 48 hours of transfection, a dual-luciferase reporter assay system (Promega) was used to measure the luciferase activity of firefly luciferase.

### 2.12. Statistical Analysis

The data were shown as the mean (SD). Data with only two groups were analyzed by Student's *t*-test, while differences among multiple groups were analyzed by one-way analysis of variance using SPSS 22.0 statistical software (IBM, Armonk, New York, USA) followed by the post hoc Bonferroni test. The differences were considered to be statistically significant when *p* < 0.05.

## 3. Results

### 3.1. SQC Improved the Diabetic Thoracic Aortic Complications

**Figure 1:**
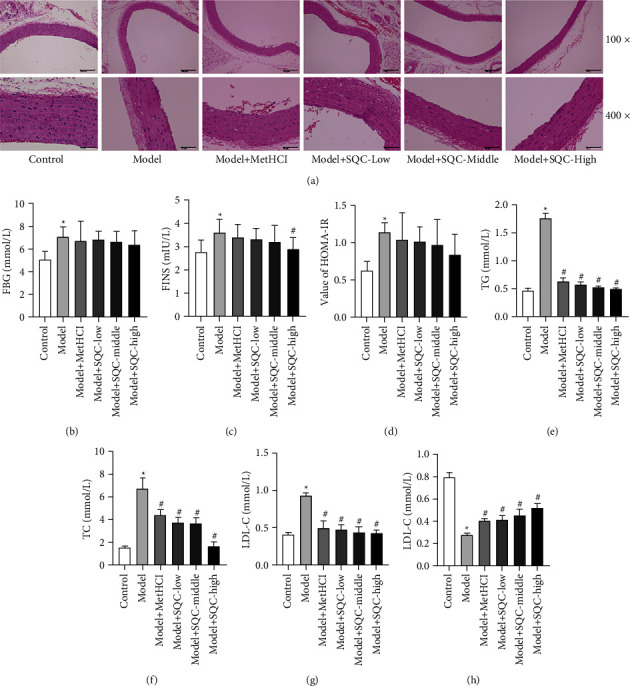
SQC relieved the diabetic thoracic aortic complications. (a) The pathological manifestations were assessed by H&E staining. (b and c) The serum levels of FBG and FINS were detected using commercial kits. (d) The value of HOMA-IR was gained using the following formula: HOMA-IR = FBG × FINS/22.5. (e–h) The serum levels of TG, TC, LDL-C, and HDL-C were examined using an automatic biochemical analyzer. ^*∗*^*p* < 0.05*vs.* Control group. ^#^*p* < 0.05*vs.* Model group. All assays were performed six times.

To determine the effect of SQC on diabetic thoracic aortic disease, a high-fat diet was administered to the model for four weeks. The structure and morphology of the intima of the aorta were observed under the microscope after H&E staining. It was confirmed that the inner wall of the aorta was covered by a single layer of cells called endothelial cells, and these endothelial cells were found to have a flattened appearance. Compared with rats in the control group, GK rats in the model group showed thinner intima, tumidness of a small number of endothelial cells, enlarged nuclei, exfoliation of part of the endothelial cells, rough surface, many endothelial cells with vacuolar degeneration, cytoplasmic vacuolation, the existence of a few elastic fibers and smooth muscle fibers in the media layer, and the thicker adventitia layer containing connective tissue and longitudinal smooth muscle fibers (Figure 1(a)). Treatment with metformin and all three doses of SQC improved these pathological manifestations (Figure 1(a)). In addition, the serum levels of FBG and FINS in the model group rats were significantly higher than those in the control group (Figures 1(b) and 1(c)). The administration of metformin and all three dosages of SQC had no statistically significant effect on FBG serum levels compared with a model group. While the high dose of SQC markedly reduced the serum levels of FINS and treatment of metformin, low and middle doses of SQC also only declined the serum levels of FINS with no statistical difference compared with those in the model group (Figures 1(b) and 1(c)). Consequently, the value of HOMA-IR in the model group was likewise significantly increased compared with the control group, and this enhancement was attenuated by metformin and all three doses of SQC (Figure 1(d)). Moreover, blood biochemistry results revealed that the levels of TG, TC, and LDL-C were prominently increased compared with those in the model group (Figures 1(e)–1(g)), which were notably antagonized with the administration of metformin and all three doses of SQC. However, an inverse tendency was observed in the serum level of HDL-C (Figure 1(h)). Therefore, these results indicated that SQC ameliorated the diabetic thoracic aortic complications.

### 3.2. SQC Relieved the Vascular Injury in Rats with Diabetic Thoracic Aortic Complications

**Figure 2:**
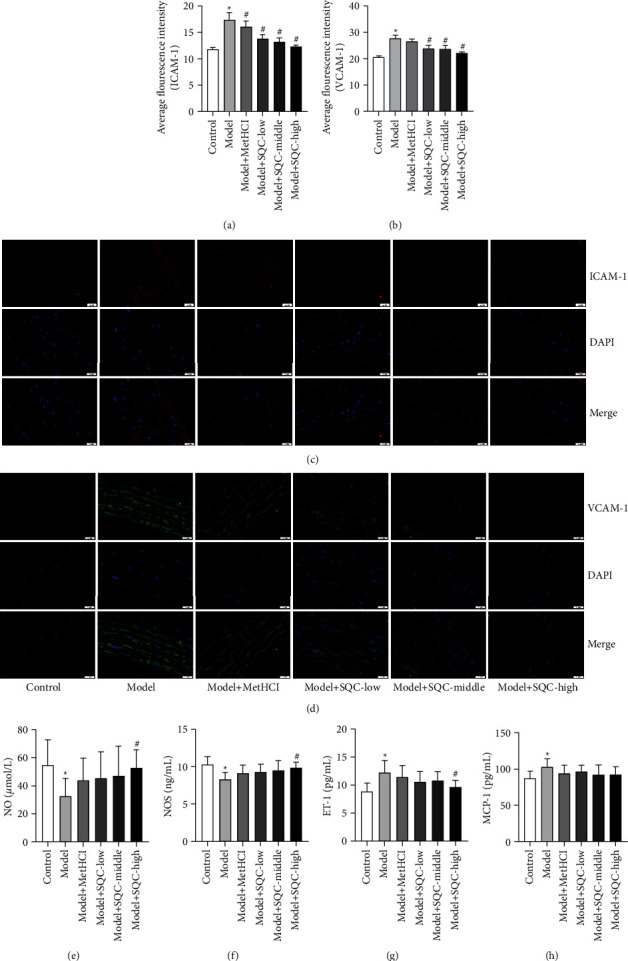
SQC mitigated the vascular injury in rats with diabetic thoracic aortic complications. (a–d) The average fluorescence intensity of ICAM-1 (a and c) and VCAM-1 (b and d) was analyzed by IF. (e–h) The concentration of NO, NOS, ET-l, and MCP-1 was determined using commercial kits. ^*∗*^*p* < 0.05*vs.* Control group. ^#^*p* < 0.05*vs.* Model group. All assays were performed six times.

The effect of SQC on vascular damage in diabetic rats with thoracic aortic problems was then studied. The IF results showed that the average fluorescence intensity of ICAM-1 and VCAM-1 in the model group was notably enhanced compared with that in the control group. Administration of metformin and all three doses of SQC markedly decreased the average fluorescence intensity of ICAM-1 compared with those in the model group, while treatment with metformin and middle and high doses of SQC significantly reduced the average fluorescence intensity of VCAM-1 compared with those in the model group, and low doses of SQC only declined the average fluorescence intensity of VCAM-1 with no statistical difference compared with those in the model group (Figures 2(a)–2(d)). In addition, the concentration of NO and NOS levels in the model group was much lower than that in the control group but was observably restored by a high dosage of SQC (Figures 2(e) and 2(f)). However, the concentration of ET-l and MCP-1 in the model group was markedly increased compared with that in the control group. Treatment with high dose of SQC notably declined the concentration of ET-l, and treatment with metformin and low and middle doses of SQC also just reduced the concentration of ET-l with no statistical difference compared with that in the model group, While the addition of metformin and all three dosages of SQC lowered the concentration of MCP-1 relative to the model group, there was no statistically significant difference (Figures 2(g) and 2(h)). Thus, these results suggest that SQC alleviated the vascular injury in rats with diabetic thoracic aortic complications.

### 3.3. SQC Reduced the Apoptosis and Oxidative Stress in Rats with Diabetic Thoracic Aortic Complications

**Figure 3:**
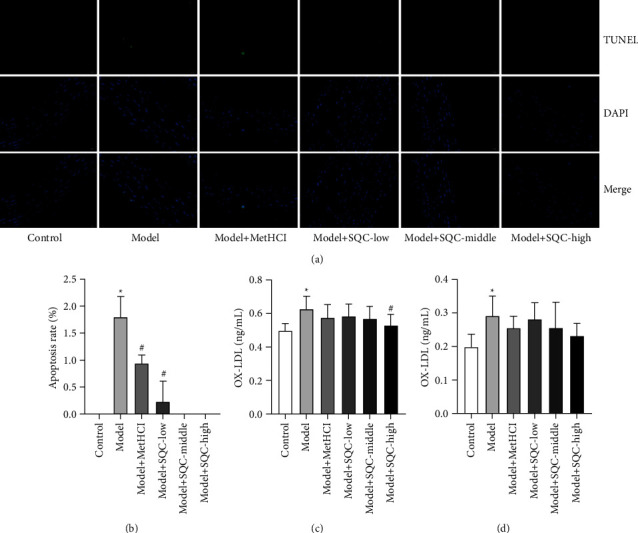
SQC decreased the apoptosis and oxidative stress in rats with diabetic thoracic aortic complications. (a and b) The apoptosis rate was detected by TUNEL staining. (c and d) The concentrations of ox-LDL in the serum and aorta were examined by ELISA. ^*∗*^*p* < 0.05*vs.* Control group. ^#^*p* < 0.05*vs.* Model group. All assays were performed six times.

Next, SQC's effect on apoptosis and oxidative stress in diabetic thoracic aortic rats was examined. TUNEL staining showed blue fluorescence in normal aortic endothelial cell nuclei and green fluorescence in apoptotic aortic endothelial cell nuclei with chromatin aggregation and concentration. The results show that administration of metformin and all three doses of SQC significantly decreased the rate of apoptosis caused by a high-fat diet (HFD) (Figures 3(a) and 3(b)). In addition, the levels of ox-LDL from the serum and aorta were considerably increased with HFD induction compared with those in the control group, which was only counteracted by metformin and all three dosages of SQC with no statistical difference (Figures 3(c) and 3(d)). Hence, these findings demonstrated that SQC decreased apoptosis and oxidative stress in rats with diabetic thoracic aortic complications.

### 3.4. SQC Promoted Macrophage Polarization and Dendritic Cell Activation in Rats with Diabetic Thoracic Aortic Complications

**Figure 4:**
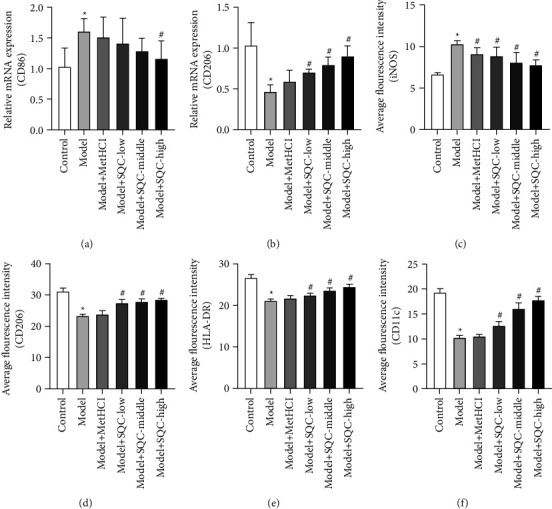
SQC promoted macrophage polarization and dendritic cell activation in rats with diabetic thoracic aortic complications. (a and b) The relative mRNA of CD86 and CD206 in the spleen of rats were measured by qRT-PCR. (c–f) The expressions of iNOS, CD206, HLA-DR, and CD11c in the thoracic aorta were determined by IF. ^*∗*^*p* < 0.05*vs.* Control group. ^#^*p* < 0.05*vs.* Model group. All assays were performed six times.

The results showed that the relative mRNA expressions of CD86 and CD206 in the spleen of rats, the marker of M1 and M2 macrophages, respectively, were markedly increased and decreased in rats fed with HFD several times (Figures 4(a) and 4(b)). Only a high dose of SQC was able to prevent the HFD-induced relative mRNA expression of CD86, but all three doses of SQC were able to more effectively rescue the HFD-induced relative mRNA expression of CD206 (Figures 4(a) and 4(b)). Besides, metformin and all three doses of SQC notably reduced the HFD-induced relative levels of iNOS, the marker of M1 macrophages, while the three doses of SQC significantly restored the HFD-induced relative levels of CD206, the marker of M2 macrophages in the thoracic aorta (Figures 4(c) and 4(d), Figure S1). Furthermore, the three dosages of SQC increased the HFD-induced relative levels of HLA-DR and CD11c in aortic dendritic cells (Figures 4(e) and 4(f), Figure S2). These findings suggested that SQC increased the polarization of macrophages and the activation of dendritic cells in rats with diabetic thoracic aortic complications.

### 3.5. SQC Regulated the Expression of Helper T Cells in Rats with Diabetic Thoracic Aortic Complications

**Figure 5:**
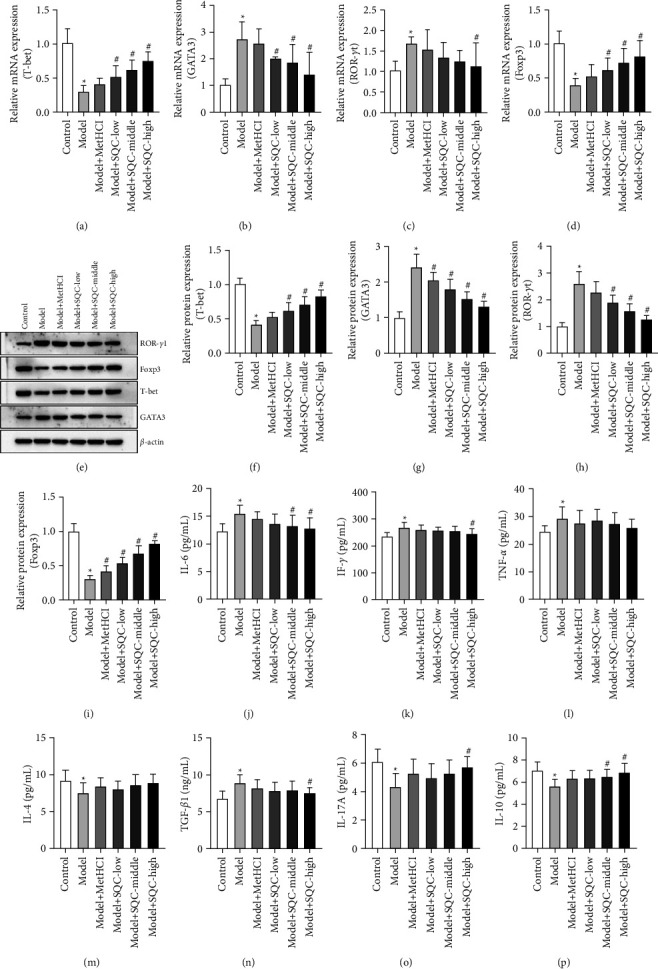
SQC regulated the expression of helper T cells in rats with diabetic thoracic aortic complications. (a–d) The relative mRNA expressions of T-bet (a), GATA3 (b), ROR-*γ*t (c), and Foxp3 (d) were detected by qRT-PCR. The gene expressions were normalized by *β*-actin. (e–i) The relative protein expressions of T-bet (a), GATA3 (b), ROR-*γ*t (c), and Foxp3 (d) were examined by western blot. The gene expressions were normalized by *β*-actin. (j–p) The concentrations of IL-6 (j), TNF-*α* (k), IFN-*γ* (l), IL-4 (m), TGF-*β*1 (n), IL-17A (o), and IL-10 (p) were measured by ELISA. ^*∗*^*p* < 0.05*vs.* Control group. ^#^*p* < 0.05*vs.* Model group. All assays were performed six times.

**Figure 6:**
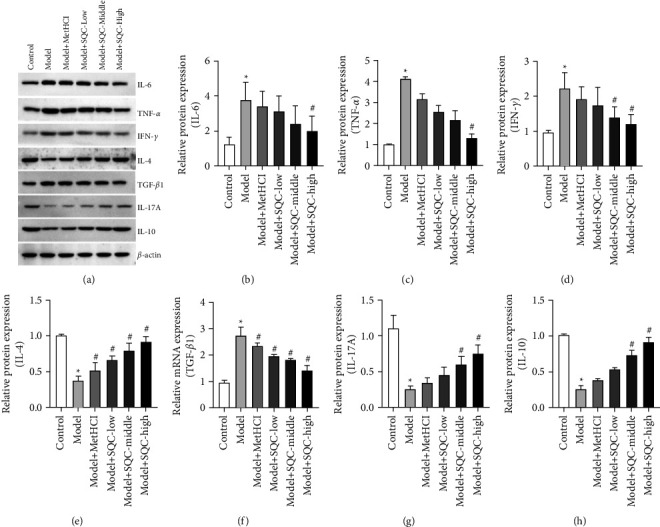
SQC modulated the protein expression of helper T cells in rats with diabetic thoracic aortic complications. (a–h) The relative protein expressions of IL-6, TNF-*α*, IFN-*γ*, IL-4, TGF-*β*1, IL-17A, and IL-10 were examined by western blot. The expressions were normalized by *β*-actin. ^*∗*^*p* < 0.05*vs.* Control group. ^#^*p* < 0.05*vs.* Model group. All assays were performed six times.

Additionally, all three doses of SQC significantly increased the HFD-induced relative mRNA expression of T-bet, whereas the same levels significantly lowered the HFD-induced relative mRNA expression of GATA3 (Figures 5(a) and 5(b)). Only high doses of SQC prominently neutralized the HFD-induced relative mRNA expression of ROR-*γ*t, whereas all three doses of SQC significantly compensated for the HFD-induced relative mRNA expression of Foxp3 (Figures 5(c) and 5(d)). Additionally, comparable patterns were seen in the relative protein expression of T-bet, GATA3, ROR-t, and Foxp3 (Figures 5(e)–5(i)). Briefly, all three dosages of SQC restored the HFD-induced relative protein expression of T-bet, but metformin and all three doses of SQC inhibited the HFD-induced relative protein expression of GATA3 (Figures 5(e)–5(g)). Metformin and all three doses of SQC also prominently counteracted the HFD-induced relative protein expression of ROR-*γ*t, while the three doses of SQC markedly rescued the HFD-induced relative protein expression of Foxp3 (Figures 5(e), 5(h), and 5(i)). Furthermore, both the middle and high doses of SQC significantly reduced HFD-induced IL-6 levels (Figure 5(j)), while the high dose of SQC also significantly reduced HFD-induced IFN-*γ* levels (Figure 5(k)). Besides, metformin and all three doses of SQC only decreased the HFD-induced concentration of TNF-*α* with no statistical difference (Figure 5(l)). On the contrary, metformin and all three doses of SQC just enhanced the HFD-induced level of IL-4 with no statistical difference (Figure 5(m)), while the high dose of SQC notably diminished the HFD-induced level of TGF-*β*1 (Figure 5(n)). In addition, only a high dose of SQC particularly raised the HFD-induced IL-17A level (Figure 5(o)), although both moderate and high doses of SQC significantly enhanced the HFD-induced IL-10 concentration (Figure 5(p)). Moreover, the effect of SQC on the expressions of IL-6, TNF-*α*, IFN-*γ*, IL-4, TGF-*β*1, IL-17A, and IL-10 was confirmed through western blot (Figure 6). Based on these data, SQC modulated the expression of helper T cells in rats with diabetic thoracic aortic complications.

### 3.6. SQC Regulated the miR-223-3p/RBP-J/IRF8 Axis in Macrophages of Rats with Diabetic Thoracic Aortic Complications

**Figure 7:**
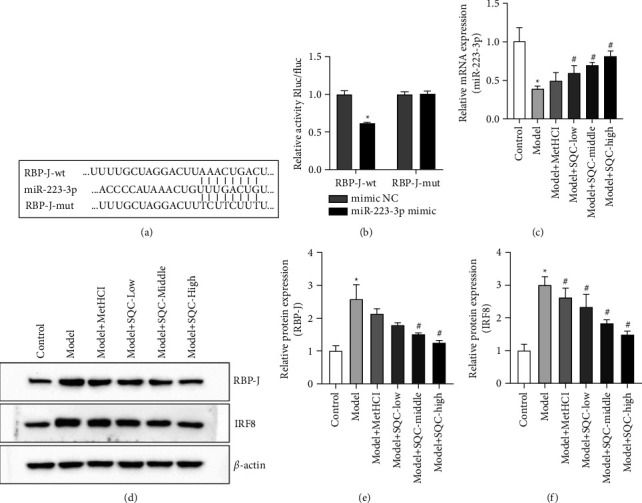
SQC mediated the miR-223-3p/RBP-J/IRF8 axis in the macrophage of rats with diabetic thoracic aortic complications. (a) The binding between miR-223-3p and RBP-J was predicted by the TargetScan website. (b) The binding between miR-223-3p and RBP-J was verified by the dual-luciferase reporter assay. (c) The level of miR-223-3p in macrophage was examined by qRT-PCR. (d–f) The relative protein expressions of RBP-J (d and e) and IRF8 (d and f) were analyzed by western blot. The gene expressions were normalized by *β*-actin. ^*∗*^*p* < 0.05*vs.* Control group. ^#^*p* < 0.05*vs.* Model group. All assays were performed six times.

The binding between miR-223-3p and RBP-J was predicted by the TargetScan website (Figure 7(a)), which was further verified by the dual-luciferase reporter assay (Figure 7(b)). Moreover, the level of miR-223-3p in macrophages of rats with diabetic thoracic aortic complications was significantly downregulated, which was notably reversed by all three doses of SQC (Figure 7(c)). The relative protein expression of RBP-J was inversely increased with HFD induction and was markedly decreased by both moderate and high dosages of SQC (Figures 7(d) and 7(e)). In addition, metformin and all three dosages of SQC reversed the HFD-induced increase in IRF8 protein expression (Figures 7(d) and 7(f)). Therefore, these data indicated that SQC modulated the miR-223-3p/RBP-J/IRF8 axis in the macrophages of rats with diabetic thoracic aortic complications.

## 4. Discussion

In this particular study, the diabetic thoracic aortic complications model was developed by providing a high-fat diet (HFD) for 4 weeks in GK rats. SQC treatment improved the HFD-induced pathological symptoms, the increased concentrations of FBG, FINS, TG, TC, and LDL-C, as well as the decreased concentrations of HDL-C, suggesting that SQC ameliorated the diabetic thoracic aortic complications. Besides, SQC counteracted the HFD-induced average fluorescence intensity of ICAM-1 and VCAM-1, as well as the concentrations of ET-l and MCP-1, while rescuing the HFD-induced concentrations of NO and NOS, indicating that SQC alleviated the vascular injury in rats with diabetic thoracic aortic complications. Also, SQC neutralized the HFD-induced apoptosis rate and the concentrations of ox-LDL from the serum and aorta, which revealed that SQC declined the apoptosis and oxidative stress in rats with diabetic thoracic aortic complications. SQC also reduced the HFD-induced relative mRNA expressions of CD86 with the enhanced relative mRNA expressions of CD206 of macrophages, decreased the HFD-induced relative levels of iNOS with the increased relative levels of CD206 in the thoracic aorta, and restored the HFD-induced relative levels HLA-DR and CD11c in aortic dendritic cells, which indicated that SQC increased the polarization of macrophages and the activation of dendritic cells in rats with diabetic thoracic aortic complications. Moreover, SQC restored the changes in both mRNA and protein expressions of T-bet, GATA3, ROR-*γ*t, and Foxp3, as well as the HFD-induced concentrations of IL-6, TNF-*α*, IFN-*γ*, IL-4, IL-17A, and IL-10, which indicated that SQC modulated the expression of helper T cells in rats with diabetic thoracic aortic complications. Furthermore, SQC also modulated the miR-223-3p/RBP-J/IRF8 axis in the macrophages of rats with diabetic thoracic aortic complications. Thus, SQC ameliorates diabetic thoracic aortic problems by regulating apoptosis, oxidative stress, and the inflammatory milieu via the miR-223-3p/RBP-J/IRF8 axis.

Apoptosis and oxidative stresses are important mechanisms involved in diabetes and its complications. Numerous studies have shown that oxidative stress plays a crucial role in diabetes [16] and its complications [17]. Ihnat et al. [18] found that large quantities of free radicals and reduced coenzyme I(II) [NAD(P)H] continued to be produced after human umbilical vein endothelial cells were cultured in a normal glucose concentration for 7 d following in a high-glucose environment for 14 d. Kowluru et al. [19] reported that higher levels of oxidative stress were present in diabetic mice with a blood sugar under control for six months, whereas the blood sugar was well controlled after five months. Thus, approaches that intervene in early hyperglycemia accompanied by the reduction of the impact of oxidative stress and its active components on the diabetic thoracic aortic complication to reduce the harmful metabolic memory caused by hyperglycemia and prevent the occurrence and development of the diabetic thoracic aortic complication are good choices for the treatment of the diabetic thoracic aortic complications [6]. In line with these findings, the present research indicated that SQC neutralized the HFD-induced amounts of ox-LDL in the serum and aorta, indicating that SQC decreased apoptosis and oxidative stress in diabetic thoracic aortic complication rats. Additionally, apoptosis is also involved in the metabolic memory triggered by high glucose. Kowluru and Chan [20] proposed that the continuous activation of the apoptosis pathway was responsible for the recurrence of diabetic retinopathy when good blood sugar management was restored, and the apoptosis of retinal cells was related with the metabolic memory of high glucose. Here, we discovered that SQC antagonized the HFD-induced apoptosis rate, which suggested that SQC declined the apoptosis in rats with diabetic thoracic aortic complications. So, SQC made the complications of diabetic thoracic aortic disease better by stopping apoptosis and oxidative stress.

An inflammatory response is an important factor causing metabolic memory phenomena in the early abnormal metabolic environment and is the target of early intervention. In the process of atherosclerosis, inflammation is one of the important pathological factors in which C-reactive protein (CRP) [21], TNF-*α* [22], IL-6 [23], and other inflammatory cytokines play an important role in the occurrence and development of large vessel atherosclerosis in diabetes. Villeneuve et al. [24] discovered that the genes of the vascular smooth muscle cells of db/db diabetic rats that encode inflammatory factors monocyte chemoattractant protein-1 (MCP-1) and IL-6 after passage are still highly expressed *in vitro*, suggesting that when the high glucose status is corrected, there is an important relationship between the persistence of inflammatory components and metabolic memory. SQC reversed the changes in both mRNA and protein expressions of T-bet, GATA3, ROR-*γ*t, and Foxp3, as well as the HFD-induced concentrations of IL-6, TNF-*α*, IFN-*γ*, IL-4, IL-17A, and IL-10. CD4^+^ T cells can differentiate into different subsets, containing Th1, Th2, Th9, Th17, Treg, and Tfh, that modulate the generation of various immune responses to regulate the elimination of diverse pathogens and immune outcomes [25]. Th1 cells upregulate T-bet through IFN-*γ* and IL-12 stimulation to trigger the production of IFN-*γ*, TNF-*α*, and IL-2; Th2 cells upregulate GATA3 by IL-4 stimulation to elicit the generation of IL-4, IL-5, and IL-13; Th17 cells upregulate ROR-*γ*t via IL-6 stimulation to evoke the production of IL-17A, IL-17F, and IL-22; Treg cells upregulate Foxp3 through TGF-*β* stimulation to induce the generation of IL-10 and TGF-*β*; and Tfh cells upregulate Bcl-6 via IL-21 and IL-6 stimulation to trigger the production of IL-21 and IL-10 [26]. Th17 cells, on the one hand, regulate the immune-inflammatory milieu in macrovascular areas by promoting angiogenesis via IL-17 and performing autoimmune suppressive effects. Moreover, Th17 cells drive anti-large vessel area immune-inflammatory responses by recruiting immune cells into large vessels, activating effector CD8(+) T cells, or even directly by converting to a Th1 phenotype and producing IFN-*γ*. In addition, it has been shown that anti-inflammatory cytokines (IL-4, IL-10) *in vivo* can inhibit the rise of proinflammatory cytokines and reduce the inflammatory response. So, the interactions and balance of proinflammatory and anti-inflammatory cytokines are very important for keeping the immune system in the body working well. In the present study, the dynamic balance of proinflammatory and anti-inflammatory cytokines in HFD-induced rats was disrupted, which was manifested as dysregulation of serum anti-inflammatory factors (IL-4, IL-10) and proinflammatory factors (IL-17A) in HFD-induced rats. However, SQC treatment reversed these changes. Moreover, Th cells can influence the activation and function of antigen-presenting cells, such as macrophages and DC cells. Thus, in the current study, SQC decreased the HFD-induced relative mRNA expressions of CD86 while increasing the relative mRNA expressions of CD206 in macrophages. Furthermore, SQC decreased the HFD-induced relative levels of iNOS while increasing the relative levels of CD206 in the thoracic aorta. Consequently, the HFD-induced relative levels of HLA-DR and CD11c are restored in aortic dendritic cells. These results indicate that SQC promoted macrophage polarization and dendritic cell activation in rats with diabetic thoracic aortic complications, also consistent with the level of the downstream immune cells. In the current study, however, the data revealed that SQC improved the consequences of diabetic thoracic aortic disease by regulating the inflammatory microenvironment.

MiR-223-3p has been demonstrated to act as a potential biomarker and molecule for pre-onset dysfunction in type 2 diabetes, of which low levels of miR-223-3p are associated with impaired insulin sensitivity [27]. In addition, miR-223-3p plays an important role in the occurrence and development of cardiovascular diseases [28], and miR-223-3p is also considered to be a bone marrow-specificmiRNA-affecting hematopoiesis, immune response, and inflammatory diseases [29–32]. More importantly, the miR-223 signaling axis is involved in the regulation of macrophage-mediated inflammation [33]. Therefore, miR-223-3p may become a valuable new target for assessing the early risk of type 2 diabetes in clinical practice and improving metabolic memory in the immune-inflammatory microenvironment of macrovascular regions [34]. Additionally, the RBP-J/IRF8 signaling pathway is related to macrophage polarization that can enhance the TLR4-induced expression of key mediators in M1 macrophages and selectively promote the synthesis of IRF8. Loss of RBP-J can downregulate the level of M1 macrophage marker iNOS and inflammatory factors, while IRF8 regulates macrophage polarization by participating in the regulation of downstream M1 macrophage gene expression. Therefore, activation of RBP-J/IRF8 signaling plays a crucial role in regulating the immune-inflammatory microenvironment. In the present study, the binding between miR-223-3p and RBP-J was predicted by the TargetScan website, which was further verified by the dual-luciferase reporter assay. Moreover, SQC notably restored the HFD-induced level of miR-223-3p in the macrophages of rats with diabetic thoracic aortic complications. Furthermore, SQC observably inverted the HFD-induced increase of the relative protein expression of RBP-J and IRF8. Therefore, the underlined results suggested that SQC regulated the miR-223-3p/RBP-J/IRF8 axis in the macrophages of diabetic thoracic aortic complications-afflicted rats.

Taken together, the findings of this study showed that SQC reduced diabetic thoracic aortic complications by modulating apoptosis, oxidative stress, and the inflammatory microenvironment via the miR-223-3p/RBP-J/IRF8 axis. However, this study has some limitations. Although miR-223-3p is reported to serve as a sponger of long noncoding RNAs or circular RNAs in a wide variety of disease models, several studies have demonstrated that miR-223-3p can be regulated by the extraction of Chinese herbal medicine. For instance, nanocurcumin dampens insulin resistance and autophagy flare with improved *β* cell mass via regulating miR-223-3p and NF-*κ*B levels in the pancreas of a rat model of polycystic ovary syndrome [35]. Icariin regulates the miR-223-3p/NLRP3 signaling axis to relieve rheumatoid arthritis [36]. Sinomenine attenuates cartilage degeneration through modulating miR-223-3p/NLRP3 inflammasome signaling [37]. SQC is an extract of eight different drugs. Each drug contains various active ingredients, and each active ingredient corresponds to many targets. Thus, an extensive study is needed to investigate the target structure of SQC for miR-233-3p, which will be the focus of our future study. Briefly, the present study provides a feasible direction for the research and development of innovative drugs.

## Figures and Tables

**Table 1:** The primer sequences used in the present study.

Name	Forward (5′-3′)	Reverse (5′-3′)
CD86	CAACGGAATTAGGAAGAC	CTCTGTATGCAAGTTTCC
CD206	CAAGGAAGGTTGGCATTTGT	CCTTTCAGTCCTTTGCAAGC
ROR-*γ*t	TGGCACCCAGCACAATGAA	CTAAGTCATAGTCCGCTAGAAGCA
Foxp3	GCACAAGTGCTTTGTGCGAGT	TGTCTGTGGTTGCAGACGTTGT
T-bet	TCCACCCAGACTCCCCCAACA	GGCTCACCGTCATTCACCTCCA
GATA3	TTCCTGTGCGAACTGTCAGACCA	CCTTTTTGCACTTTTTCGATTTGCTA
*β*-Actin	GGAGATCAAGATCATTAGTCCT	TACTCCTGCTTGCTGATCCA
miR-223-3p	GAAGCTGTACCTAACATACCGTG	GATTGGTCGTGGACGTGTCG
U6	AGTAAGCCCTTGCTGTCAGTG	CCTGGGTCTGATAATGCTGGG

## Data Availability

The data sets used and analyzed during the current study are available from the corresponding authors on reasonable request.

## References

[1] Saeedi P., Petersohn I., Salpea P. (2019). Global and regional diabetes prevalence estimates for 2019 and projections for 2030 and 2045: results from the international diabetes federation diabetes atlas. *Diabetes Research and Clinical Practice*.

[2] Chowdhury T. A., Shaho S., Moolla A. (2014). Complications of diabetes: progress, but significant challenges ahead. *Annals of Translational Medicine*.

[3] Leon B. M., Maddox T. M. (2015). Diabetes and cardiovascular disease: epidemiology, biological mechanisms, treatment recommendations and future research. *World Journal of Diabetes*.

[4] Writing Team for the Diabetes Control and Complications Trial/Epidemiology of Diabetes Interventions and Complications Research Group (2003). Sustained effect of intensive treatment of type 1 diabetes mellitus on development and progression of diabetic nephropathy: the Epidemiology of Diabetes Interventions and Complications (EDIC) study. *JAMA*.

[5] Dluhy R. G., McMahon G. T. (2008). Intensive glycemic control in the ACCORD and ADVANCE trials. *New England Journal of Medicine*.

[6] Ceriello A., Ihnat M. A., Thorpe J. E. (2009). Clinical review 2: the “metabolic memory”: is more than just tight glucose control necessary to prevent diabetic complications?. *Journal of Clinical Endocrinology and Metabolism*.

[7] Donath M. Y., Shoelson S. E. (2011). Type 2 diabetes as an inflammatory disease. *Nature Reviews Immunology*.

[8] Cipolletta D. (2014). Adipose tissue-resident regulatory T cells: phenotypic specialization, functions and therapeutic potential. *Immunology*.

[9] Fang Z. H., Ni Y. Q. (2008). *Intervention Effect of Danzhijiangtang Capsule on Vascular Endothelium Injury Induced by Oxidative Stress in Type Two Diabetes Mellitus*.

[10] Fang Z. H. (2008). The clinical observation of Yiqiyangyinhuoxue therapy on type 2 diabetes’ vascular endothelium injury induced by adhesion condition and inflammation. *Chinese medicine emergency*.

[11] Chen Q. Y. (2013). Clinical observation on 60 cases of diabetic vascular disease treated by danqi dendrobium recipe. *Practical clinical practice of integrated traditional Chinese and western medicine*.

[12] Wang H. (2020). Intervention research on vascular endothelial injury in prethrombotic state in patients with type 2 diabetes mellitus by nourishing qi, nourishing yin and activating blood. *Oriental medicinal diet*.

[13] Hu Y. H. (2014). Protective effect and mechanism of Shenqi compound on vascular lesions in diabetic model rats. *Chinese Journal of Integrative Medicine*.

[14] Yang Q. Y. (2014). Study on the mechanism of Shenqi compound in preventing and treating diabetic macrovascular disease. *Chinese herbal medicine*.

[15] Fu X. X. Exploration on the molecular mechanism of nourishing yin, nourishing qi and activating blood to block “metabolic memory. *Diabetic GK Rats*.

[16] Szabo C. (2009). Role of nitrosative stress in the pathogenesis of diabetic vascular dysfunction. *British Journal of Pharmacology*.

[17] de M Bandeira S., da Fonseca L., da S Guedes G., Rabelo L., Goulart M., Vasconcelos S. (2013). Oxidative stress as an underlying contributor in the development of chronic complications in diabetes mellitus. *International Journal of Molecular Sciences*.

[18] Ihnat MA., Thorpe J. E., Ceriello A. (2009). Hypothesis: the “metabolic memory,” the new challenge of diabetes. *Diabetes Research and Clinical Practice*.

[19] Kowluru R. A., Abbas S. N., Odenbach S. (2004t). Reversal of hyperglycemia and diabetic nephropathy: effect of reinstitution of good metabolic control on oxidative stress in the kidney of diabetic rats. *Journal of Diabetes and Its Complications*.

[20] Kowluru R. A., Chan P. S. (2010). Metabolic memory in diabetes—from in vitro oddity to in vivo problem: role of apoptosis. *Brain Research Bulletin*.

[21] Gotto A. M. (2007). Role of C-reactive protein in coronary risk reduction: focus on primary prevention. *The American Journal of Cardiology*.

[22] Zhang H., Park Y., Wu J. (2009). Role of TNF-alpha in vascular dysfunction. *Clinical Science*.

[23] Aso Y., Okumura K., Yoshida N. (2003). Plasma interleukin-6 is associated with coagulation in poorly controlled patients with Type 2 diabetes. *Diabetic Medicine*.

[24] Villeneuve L. M., Reddy M. A., Lanting L. L., Wang M., Meng L., Natarajan R. (2008). Epigenetic histone H3 lysine 9 methylation in metabolic memory and inflammatory phenotype of vascular smooth muscle cells in diabetes. *Proceedings of the National Academy of Sciences of the United States of America*.

[25] O’Shea J. J., Paul W. E. (2010). Mechanisms underlying lineage commitment and plasticity of helper CD4+ T cells. *Science*.

[26] Saravia J., Chapman N. M., Chi H. (2019). Helper T cell differentiation. *Cellular and Molecular Immunology*.

[27] Sánchez-Ceinos J., Rangel-Zuñiga O. A., Clemente-Postigo M. (2021). miR-223-3p as a potential biomarker and player for adipose tissue dysfunction preceding type 2 diabetes onset. *Molecular Therapy—Nucleic Acids*.

[28] Zhang M. W., Shen Y. J., Shi J., Yu J. G. (2020). MiR-223-3p in cardiovascular diseases: a biomarker and potential therapeutic target. *Frontiers in Cardiovascular Medicine*.

[29] Chen C. Z., Li L., Lodish H. F., Bartel D. P. (2004). MicroRNAs modulate hematopoietic lineage differentiation. *Science*.

[30] Johnnidis J. B., Harris M. H., Wheeler R. T. (2008). Regulation of progenitor cell proliferation and granulocyte function by microRNA-223. *Nature*.

[31] Aziz F. (2016). The emerging role of miR-223 as novel potential diagnostic and therapeutic target for inflammatory disorders. *Cellular Immunology*.

[32] Haneklaus M., Gerlic M., O’Neill L. A. J., Masters S. L. (2013). miR-223: infection, inflammation and cancer. *Journal of Internal Medicine*.

[33] Kim G. D., Ng H. P., Patel N., Mahabeleshwar G. H. (2019). Kruppel-like factor 6 and miR-223 signaling axis regulates macrophage-mediated inflammation. *The FASEB Journal*.

[34] Jiménez-Lucena R., Rangel-Zúñiga O. A., Alcalá-Díaz J. F. (2018). Circulating miRNAs as predictive biomarkers of type 2 diabetes mellitus development in coronary heart disease patients from the CORDIOPREV study. *Molecular Therapy—Nucleic Acids*.

[35] Abuelezz N. Z., E Shabana M., Rashed L., Nb Morcos G (2021). Nanocurcumin modulates miR-223-3p and NF-*κ*B levels in the pancreas of rat model of polycystic ovary syndrome to attenuate autophagy flare, insulin resistance and improve ß cell mass. *Journal of Experimental Pharmacology*.

[36] Wu Z. M., Luo J., Shi X. D., Zhang S. X., Zhu X. B., Guo J. (2020). Icariin alleviates rheumatoid arthritis via regulating miR-223-3p/NLRP3 signalling axis. *Autoimmunity*.

[37] Dong H. C., Li P. N., Chen C. J. (2019). Sinomenine attenuates cartilage degeneration by regulating miR-223-3p/NLRP3 inflammasome signaling. *Inflammation*.

